# Acquisition of lipid metabolic capability in hepatocyte-like cells directly induced from mouse fibroblasts

**DOI:** 10.3389/fcell.2014.00043

**Published:** 2014-08-25

**Authors:** Shizuka Miura, Atsushi Suzuki

**Affiliations:** ^1^Division of Organogenesis and Regeneration, Medical Institute of Bioregulation, Kyushu UniversityFukuoka, Japan; ^2^Core Research for Evolutional Science and Technology, Japan Science and Technology AgencySaitama, Japan

**Keywords:** liver, hepatocyte, lipid metabolism, iHep cell, reprogramming, fibroblast

## Abstract

Recently, the numbers of patients with non-alcoholic fatty liver disease (NAFLD) and non-alcoholic steatohepatitis (NASH) have increased worldwide. NAFLD and NASH are known as risk factors for liver cirrhosis and hepatocellular carcinoma. Because many factors can promote the progression of NAFLD and NASH, the treatment of these patients involves various strategies. Thus, it is desired that drugs for patients with NAFLD and NASH should be developed more easily and rapidly using cultures of primary hepatocytes. However, it is difficult to use hepatocytes as a tool for drug screening, because these cells cannot be functionally maintained in culture. Thus, in this study, we sought to examine whether induced hepatocyte-like (iHep) cells, which were directly induced from mouse dermal fibroblasts by infection with a retrovirus expressing *Hnf4α* and *Foxa3*, possess the potential for lipid metabolism, similar to hepatocytes. Our data showed that iHep cells were capable of synthesizing lipids from a cis-unsaturated fatty acid, a trans-unsaturated fatty acid, and a saturated fatty acid, accumulating the synthesized lipids in cellular vesicles, and secreting the lipids into the culture medium. Moreover, the lipid synthesis in iHep cells was significantly inhibited in cultures with lipid metabolism improvers. These results demonstrate that iHep cells could be useful not only for screening of drugs for patients with NAFLD and NASH, but also for elucidation of the mechanisms underlying hereditary lipid metabolism disorders, as an alternative to hepatocytes.

## Introduction

The recent increase of the number of patients with non-alcoholic fatty liver disease (NAFLD), which often develops through obesity and lipid metabolism disorders, is an issue that has become problematic in liver diseases (Vuppalanchi and Chalasani, 2009). NAFLD can be characterized into simple steatosis and non-alcoholic steatohepatitis (NASH). In particular, NASH is associated with the risk of inducing not only liver cirrhosis and hepatocellular carcinoma, but also heart diseases (Hashimoto et al., 2009; Vuppalanchi and Chalasani, 2009). In general, as the cause of NASH developing from fatty liver disease, a “two-hit hypothesis” has been proposed (Day and James, 1998). The first hit is the onset of steatohepatitis, which is induced as a result of cellular lipid accumulation and steatosis. The second hit includes activation of inflammatory cytokines and hepatic oxidative stress (Day and James, 1998; Dowman et al., 2010). As the current therapeutic drugs for NASH, insulin-resistance improvers and antioxidants are frequently used. However, there are many factors that can promote the progression of NAFLD and NASH. Therefore, if we are able to develop many drugs for patients with NAFLD and NASH more easily and rapidly it will become possible to beat the causative factors of these diseases specifically.

Ideally, primary hepatocytes should be used in the screening of drugs for patients with liver diseases. However, it is difficult to maintain the function of hepatocytes in culture. Thus, liver cancer cell lines are often substituted for hepatocytes, although the nature of these cell lines differs from that of normal hepatocytes. To obtain functional hepatocytes for the screening of drugs, hepatocyte-like cells can be induced from embryonic stem (ES) cells and induced pluripotent stem (iPS) cells (Basma et al., 2009; Rashid et al., 2010; Si-Tayeb et al., 2010; Cayo et al., 2012; Choi et al., 2013). Moreover, recent advances in the induction of cellular reprogramming have enabled the generation of hepatocyte-like cells directly from fibroblasts, which is termed “direct reprogramming” (Huang et al., 2011; Sekiya and Suzuki, 2011). In our previous paper, we reported that three specific combinations of two transcription factors, comprising Hnf4α plus Foxa1, Foxa2, or Foxa3, can be used to convert mouse fibroblasts into hepatocyte-like cells (Sekiya and Suzuki, 2011). The induced hepatocyte-like (iHep) cells exhibited multiple hepatocyte-specific functions and restored hepatic tissues after transplantation. Thus, it is expected that iHep cells could be used for developing regenerative therapies and examining the pharmacological effects of drugs. Although iHep cells may be useful in the screening of drugs for patients with NAFLD and NASH, the capability for lipid metabolism in iHep cells remains to be clarified. Thus, in this study, we examined whether iHep cells possess the potential for lipid metabolism, similar to hepatocytes, and further attempted to clarify whether iHep cells could be useful not only for the screening of drugs but also for the progression of basic research for patients with liver diseases involving lipid metabolism.

## Materials and methods

### Generation of iHEP cells

We generated iHep cells from mouse dermal fibroblasts (MDFs) and conducted co-immunofluorescence staining of albumin with E-cadherin, as described previously (Sekiya and Suzuki, 2011). Some modifications that were added to the methods are specifically described below. In the preparation of MDFs, we incubated small pieces of skin tissue obtained from C57BL/6 adult mice (8–10 weeks of age) in Hanks' balanced salt solution containing 0.05% collagenase (Wako, Osaka, Japan) and 0.01% trypsin inhibitor (Nacalai Tesque, Kyoto, Japan) for 20 min at 37°C. The treated tissues were then collected by centrifugation (200 g for 3 min), plated on gelatin-coated six-well plates, and grown until expanded MDFs reached confluency. In the production of recombinant retroviruses, we used PLAT-E cells (Morita et al., 2000) for transfection of plasmid DNA. In this study, we used iHep cells at passage 9–12 for examination.

### Induction of lipid synthesis and secretion

To induce lipid synthesis, iHep cells were cultured in our hepato-medium (Sekiya and Suzuki, 2011) containing 20 ng/ml hepatocyte growth factor (HGF) (Sigma-Aldrich, St. Louis, MO, USA), 20 ng/ml epithelial growth factor (EGF) (Sigma-Aldrich), and 5% bovine serum albumin (BSA) (Wako) for 3 days with or without 1 mM oleic acid (Sigma-Aldrich), 1 mM elaidic acid (Tokyo Chemical Industry, Tokyo, Japan), or 1 mM palmitic acid (Wako). Hepatocytes isolated from adult mice by two-step collagenase digestion (Seglen, 1979) were also cultured under the same conditions used for iHep cells, except that HGF and EGF were omitted. MDFs were cultured in Dulbecco's modified Eagle's medium containing 10% fetal bovine serum, 2 mM L-glutamine (Nacalai Tesque), penicillin/streptomycin (Nacalai Tesque), and 5% BSA for 3 days with or without 1 mM oleic acid. To induce lipid secretion, iHep cells were cultured in phenol red-free hepato-medium containing 20 ng/ml HGF, 20 ng/ml EGF, and 5% BSA for 48 h with or without 1 mM oleic acid. The culture medium was then changed to remove the oleic acid, and the supernatants were collected at 12, 24, and 36 h after changing the culture medium.

### Oil red O staining

Cultured cells were fixed in 10% formalin for 10 min at room temperature, washed with phosphate-buffered saline (PBS), and treated with 60% isopropanol for 1 min at room temperature. The cells were then incubated with 60% Oil red O (Muto Pure Chemicals, Tokyo, Japan) in water for 20 min at room temperature. After washing with 60% isopropanol for 1 min and additional washing with PBS, the cells were incubated with hematoxylin (Muto Pure Chemicals) for 5 min.

### Measurement of triglyceride and apolipoprotein B (apo B)

Cultured cells were washed with PBS, collected using a cell scraper into a triglyceride assay buffer [5% Triton X-100 (Nacalai Tesque), 1 mM EDTA (Nacalai Tesque), and 25 mM Tris-HCl pH 7.5], and lysed by sonication. The triglyceride contents in the cell lysates and supernatants and the apo B contents in the supernatants were detected using a Triglyceride Assay Kit (BioAssay Systems, Hayward, CA, USA) and a Total Human Apo B ELISA Assay Kit (ALerCHEK Inc., Portland, ME, USA), respectively, according to the manufacturer's instructions. The absorbance signals were measured with a Multiskan FC microplate reader (Thermo Fisher Scientific, Waltham, MA, USA).

### Analysis of the effects of lipid metabolism improvers

iHep cells were cultured in hepato-medium containing 20 ng/ml HGF, 20 ng/ml EGF, and 5% BSA for 3 days with or without 1 mM oleic acid, 15 μM 5-(tetradecyloxy)-2-furoic acid (TOFA) (Sigma-Aldrich), and 10 μM clofibrate (Tokyo Chemical Industry). Hepatocytes were also cultured under the same conditions used for iHep cells, except that HGF and EGF were omitted. We added clofibrate to the culture media every day.

### Statistical analysis

Data are shown as mean ± standard deviation (SD), and statistical significance was analyzed using an unpaired Student's *t*-test or a One-Way ANOVA. *P* < 0.05 was considered statistically significant.

### Study approval

The experiments were approved by the Kyushu University Animal Experiment Committee, and the care of the animals was in accordance with institutional guidelines.

## Results

### iHEP cells can synthesize lipids from a cis-unsaturated fatty acid, similar to hepatocytes

**Figure 1:**
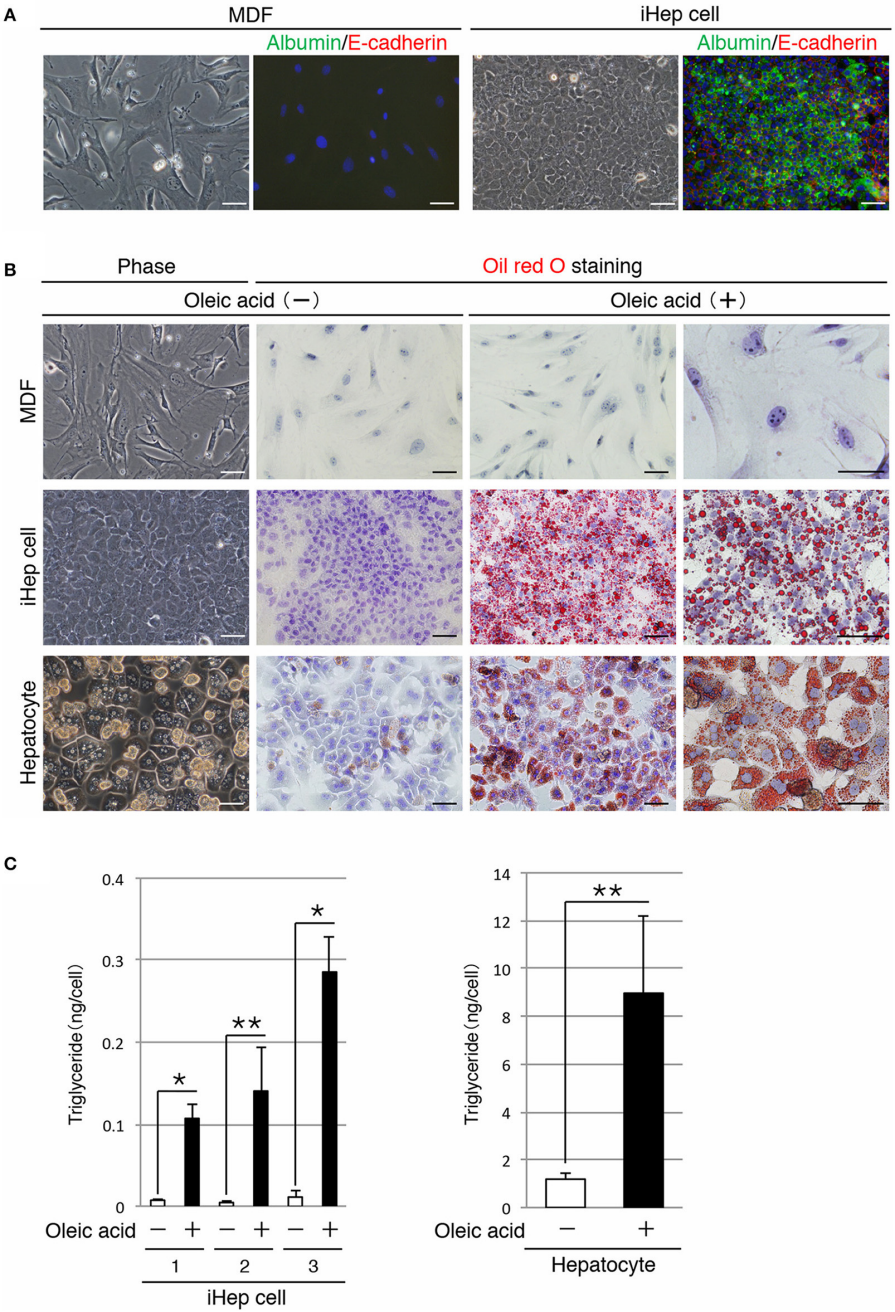
**iHep cells can synthesize lipids from a cis-unsaturated fatty acid**. **(A)** Morphologies of MDFs and MDF-derived iHep cells. Co-immunofluorescence staining of albumin and E-cadherin was conducted for MDFs and iHep cells. DNA was stained with DAPI. **(B)** iHep cells can synthesize and store abundant lipids in cultures containing oleic acid, a cis-unsaturated fatty acid. Phase-contrast images of MDFs, iHep cells, and primary hepatocytes in cultures without oleic acid are shown. Oil red O staining was conducted for MDFs, iHep cells, and primary hepatocytes cultured with (+) or without (−) oleic acid. DNA was stained with hematoxylin. **(C)** Quantification of triglyceride contents in iHep cells and primary hepatocytes cultured with (+) or without (−) oleic acid. We chose three independent iHep cells for examination. The graphs show the average of three independent experiments (mean ± SD). ^*^P < 0.01, ^**^P < 0.05 (Student's *t*-test). Scale bars, 50 μm.

In a previous study, we reported a method for the conversion of MDFs into iHep cells (Sekiya and Suzuki, 2011). In accordance with this method, we generated iHep cells by introducing *Hnf4α* and *Foxa3* into dermal fibroblasts obtained from adult mice. As shown in Figure 1A, iHep cells had features of epithelial cells and hepatocytes, including the expression of E-cadherin and albumin, respectively. iHep cells became attached to each other through intercellular adhesion molecules, and the size of the iHep cells was smaller than that of MDFs. To investigate the function of lipid metabolism in iHep cells, we used iHep cells that were generated in three independent experiments (*n* = 3). In the presence of oleic acid, a cis-unsaturated fatty acid, in the culture medium, many lipid droplets were observed in the cytoplasm of iHep cells and primary hepatocytes, but not in the cytoplasm of MDFs (Figure 1B). Moreover, the amounts of triglyceride in iHep cells and primary hepatocytes were significantly increased in cultures with oleic acid (Figure 1C). These data demonstrated that iHep cells acquired the ability to synthesize lipids from a cis-unsaturated fatty acid and accumulate these lipids into many cellular vesicles, similar to hepatocytes, by reprogramming of the original fate of fibroblasts into the hepatic fate.

### iHEP cells can synthesize lipids from a trans-unsaturated fatty acid and a saturated fatty acid

**Figure 2:**
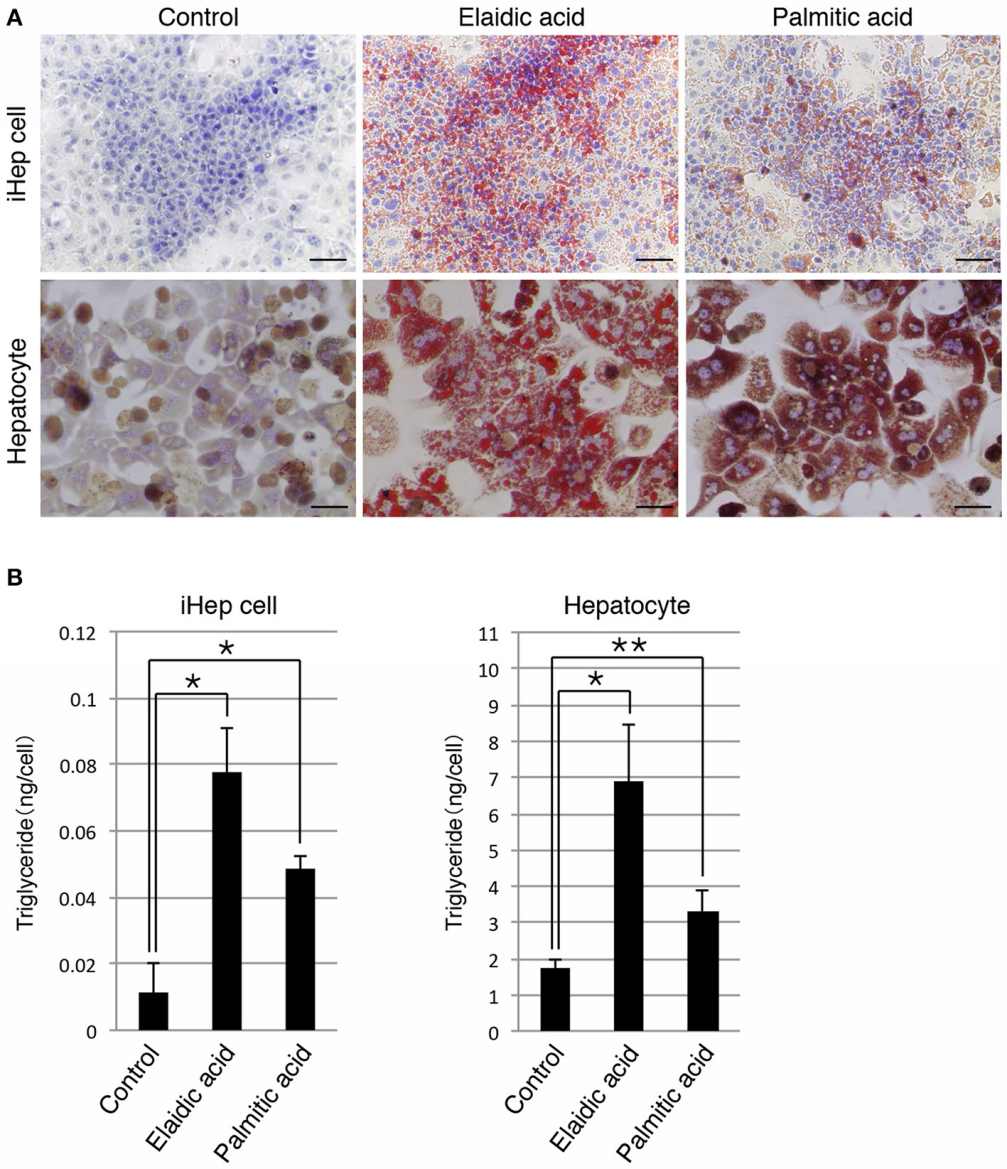
**iHep cells are capable of synthesizing lipids from a trans-unsaturated fatty acid and a saturated fatty acid. (A)** Oil red O staining was conducted for iHep cells and primary hepatocytes cultured with elaidic acid, a trans-unsaturated fatty acid, or palmitic acid, a saturated fatty acid, and without any fatty acids (control). DNA was stained with hematoxylin. Scale bars, 50 μm. **(B)** Quantification of triglyceride contents in iHep cells and primary hepatocytes cultured with elaidic acid or palmitic acid and without any fatty acids (control). The graphs show the average of three independent experiments (mean ± SD). ^*^P < 0.01, ^**^P < 0.05 (One-Way ANOVA).

In addition to a cis-unsaturated fatty acid, we examined whether iHep cells could synthesize lipids from a trans-unsaturated fatty acid and a saturated fatty acid, both of which are often present in foods. iHep cells and primary hepatocytes were each cultured with elaidic acid, a trans-unsaturated fatty acid, or palmitic acid, a saturated fatty acid, for 3 days, and then subjected to oil red O staining to investigate their lipid synthesis and accumulation. The data showed that many lipid droplets were formed in both types of cells, and that the sizes of the droplets in cells cultured with elaidic acid were larger than those in cells cultured with palmitic acid (Figure 2A). In addition, the amounts of triglyceride in iHep cells and primary hepatocytes were increased in cultures with elaidic acid or palmitic acid (Figure 2B). Thus, similar to hepatocytes, iHep cells can synthesize lipids from a trans-unsaturated fatty acid and a saturated fatty acid, and accumulate these lipids into many droplets in cells, similar to the case for the culture with a cis-unsaturated fatty acid.

### iHEP cells possess the capability for lipid secretion

**Figure 3:**
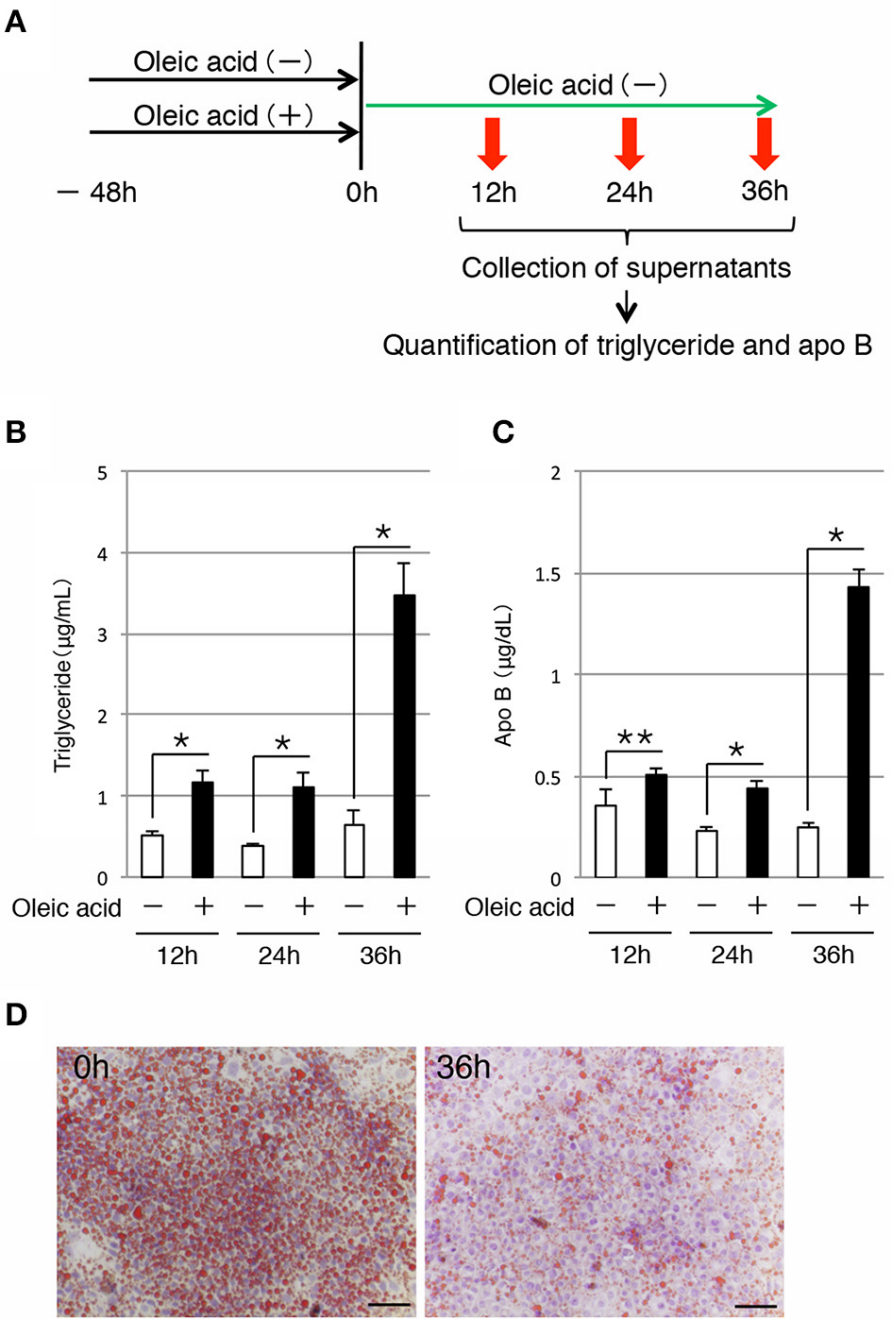
**iHep cells have the ability to secrete triglyceride and apo B. (A)** Schematic diagram of the experimental procedure. iHep cells were cultured with or without oleic acid for 48 h, and then cultured for 36 h after changing the culture medium. The supernatants were collected at 12, 24, and 36 h after changing the culture medium, and the amounts of triglyceride and apo B in the supernatants were measured. **(B,C)** Quantification of triglyceride **(B)** and apo B **(C)** contents in the supernatants collected at 12, 24, and 36 h after changing the culture medium. The amounts of triglyceride and apo B in the supernatants were normalized to the values per 1 × 10^4^ cultured cells. The graphs show the average of three independent experiments (mean ± SD). ^*^P < 0.01, ^**^P < 0.05 (Student's *t*-test). **(D)** Oil red O staining was conducted for iHep cells at 0 and 36 h after changing the culture medium. DNA was stained with hematoxylin. Scale bars, 50 μm.

Next, we examined whether iHep cells could secrete lipids to the culture medium, similar to hepatocytes. To this end, we maintained iHep cells in culture with oleic acid for 48 h, and then changed the culture medium to remove the oleic acid. The supernatants were collected at 12, 24, and 36 h after changing the culture medium for analysis (Figure 3A). Quantification of the triglyceride contents showed that the amounts of triglyceride in the supernatants increased after removal of oleic acid from the culture medium (Figure 3B). In addition to triglyceride, the amounts of apo B, which is the major protein component of triglyceride-rich lipoproteins, in the supernatants also increased after removal of oleic acid from the culture medium (Figure 3C). Moreover, the number of lipid droplets in iHep cells was decreased at 36 h after changing the culture medium, suggesting that the lipids synthesized from oleic acid in iHep cells were secreted from the cells into the supernatant (Figure 3D). These data demonstrated that iHep cells possessed the ability to not only synthesize lipids from fatty acids, but also secrete these lipids by exocytosis, similar to hepatocytes.

### Inhibition of lipid synthesis in iHEP cells by lipid metabolism improvers

**Figure 4:**
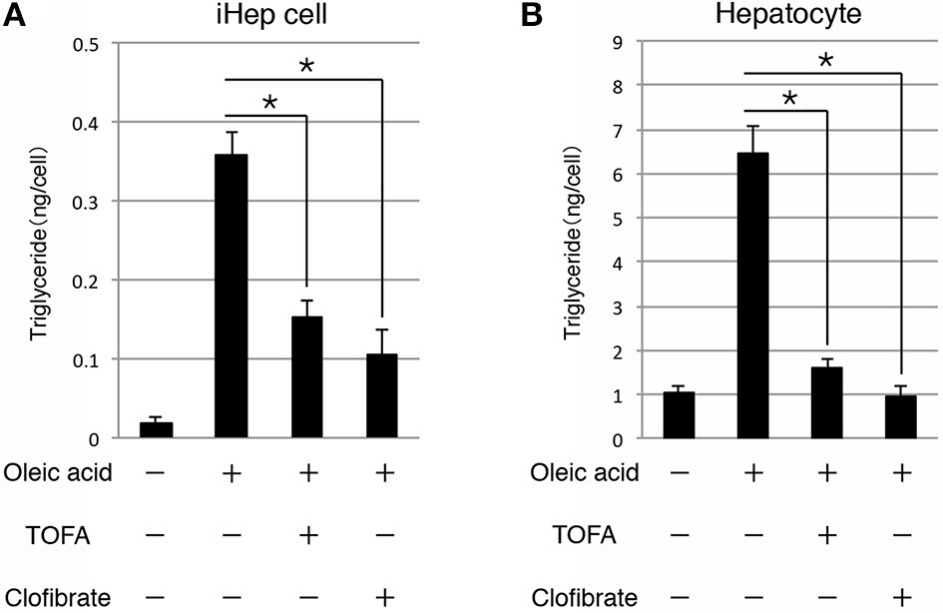
**iHep cells can respond to lipid metabolism improvers. (A,B)** Quantification of triglyceride contents in iHep cells **(A)** and primary hepatocytes **(B)** cultured with or without oleic acid, and supplemented with or without the well-known lipid metabolism improvers TOFA or clofibrate. The graphs show the average of three independent experiments (mean ± SD). ^*^P < 0.01 (One-Way ANOVA).

As shown above, iHep cells had the potential to synthesize, accumulate, and secrete lipids in culture, similar to hepatocytes, suggesting that iHep cells could be used in the screening of lipid metabolism improvers for patients with liver diseases involving lipid metabolism. Thus, we further examined whether the lipid synthesis in iHep cells could be inhibited by the effects of well-known lipid metabolism improvers. To this end, we cultured iHep cells with TOFA or clofibrate, both of which are known to act as lipid metabolism improvers, in addition to oleic acid. TOFA blocks lipid synthesis by inhibiting acetyl-CoA carboxylase in the fatty acid biosynthetic pathway, as shown in rat hepatocytes *in vitro* (Panek et al., 1977; McCune and Harris, 1979). Clofibrate is an agonist of peroxisome proliferator-activated receptor α and stimulates peroxisomal β-oxidation (Scotto et al., 1995; Wheelock et al., 2007). Both TOFA and clofibrate are used for the treatment of fatty liver diseases in humans. Our data showed that the amounts of triglyceride in iHep cells and primary hepatocytes were decreased in cultures with oleic acid and TOFA or clofibrate, indicating that iHep cells could respond to the lipid metabolism improvers and reduce lipid synthesis, similar to hepatocytes (Figure 4). Thus, iHep cells could be used for evaluating the effects of lipid metabolism improvers and screening of drugs for patients with lipid metabolism disorders, as an alternative to hepatocytes.

## Discussion

In this study, we investigated the potential for lipid metabolism in iHep cells that were directly induced from MDFs by infection with a retrovirus expressing *Hnf4α* and *Foxa3*. We found that iHep cells were not only able to synthesize lipids from a cis-unsaturated fatty acid, but also able to accumulate and secrete the synthesized lipids, similar to hepatocytes. In addition, iHep cells were capable of synthesizing lipids from a trans-unsaturated fatty acid and a saturated fatty acid, as well as from a cis-unsaturated fatty acid. Moreover, both iHep cells and primary hepatocytes were capable of responding to the well-known lipid metabolism improvers TOFA and clofibrate. Thus, it is possible that iHep cells will also respond to other lipid metabolism improvers, in addition to TOFA and clofibrate. Taken together, our data demonstrate that iHep cells have some of the important functions in hepatic lipid metabolism and could be useful in the screening of drugs for patients with lipid metabolism disorders.

In the present study, we measured the amounts of triglyceride to quantify the cellular lipid contents within iHep cells and primary hepatocytes. However, hepatocytes can also synthesize other types of lipid, including cholesterol. In our previous study, microarray data demonstrated that the expressions of genes involved in cholesterol metabolism in iHep cells became close to those in hepatocytes, suggesting that iHep cells are also able to synthesize cholesterol, similar to hepatocytes (Sekiya and Suzuki, 2011). In addition, iHep cells expressed genes involved in glucose and xenobiotic metabolism (Sekiya and Suzuki, 2011). Thus, it is possible to speculate that iHep cells possess the potential for glucose and xenobiotic metabolism, as well as that the potential for lipid metabolism that was demonstrated in this study.

Although the amount of triglyceride in iHep cells was less than that in primary hepatocytes, the function and number of iHep cells could be maintained in culture, unlike the case for hepatocytes. Currently, particular cell lines derived from liver cancer cells, which have some hepatic functions and can be maintained in culture, are used as alternatives to hepatocytes in the screening of drugs. Thus, these liver cancer cell lines would appear to be more useful than iHep cells. However, the nature of liver cancer cell lines is different from that of normal hepatocytes, and it has thus been desirable to identify a new type of cells that can be maintained in culture with the properties of normal hepatocytes to examine the pharmacological effects of drugs. Recently, in addition to iHep cells, hepatocyte-like cells induced from ES cells and iPS cells have been expected to be useful in the screening of drugs (Basma et al., 2009; Rashid et al., 2010; Si-Tayeb et al., 2010; Cayo et al., 2012; Choi et al., 2013). However, it has remained unclear whether these iHep cells and stem cell-derived hepatocyte-like cells have the potential for lipid metabolism, similar to hepatocytes. In this study, we clearly showed that iHep cells possess the properties of hepatocytes in lipid metabolism, by synthesizing lipids from fatty acids, accumulating the lipids in cellular vesicles, secreting the lipids into the culture medium, and responding to lipid metabolism improvers, even after expansion in culture for a relatively long period. Thus, iHep cells will be useful in the screening of drugs for patients with liver diseases involving lipid metabolism, as an alternative to hepatocytes and liver cancer cell lines. Moreover, it is expected that iHep cells will be useful in basic research to analyze the mechanisms underlying hereditary lipid metabolism disorders.

### Conflict of interest statement

The authors declare that the research was conducted in the absence of any commercial or financial relationships that could be construed as a potential conflict of interest.
